# Human parainfluenza virus type 2 hemagglutinin-neuramindase gene: sequence and phylogenetic analysis of the Saudi strain Riyadh 105/2009

**DOI:** 10.1186/1743-422X-9-316

**Published:** 2012-12-22

**Authors:** Fahad N Almajhdi, Mohamed S Alshaman, Haitham M Amer

**Affiliations:** 1 Department of Botany and Microbiology, College of Science, King Saud University, P.O. Box, 2455, Riyadh, 11451, Kingdom of Saudi Arabia; 2 Center of Excellence in Biotechnology Research, College of Science, King Saud University, 11451, Riyadh, Saudi Arabia; 3 Department of Virology, Faculty of Veterinary Medicine, Cairo University, 12211, Giza, Egypt

**Keywords:** Hemagglutinin-neuramindase, HPIV-2, Phylogenetic tree, Riyadh 105/2009, Saudi Arabia, Sequence analysis

## Abstract

**Background:**

Although human parainfluenza type 2 (HPIV-2) virus is an important respiratory pathogen, a little is known about strains circulating in Saudi Arabia.

**Findings:**

Among 180 nasopharyngeal aspirates collected from suspected cases in Riyadh, only one sample (0.56%) was confirmed HPIV-2 positive by nested RT-PCR. The sample that was designated Riyadh 105/2009 was used for sequencing and phylogenetic analysis of the most variable virus gene; the haemagglutinin-neuramindase (HN). Comparison of HN gene of Riyadh 105/2009 strain and the relevant sequences available in GenBank revealed a strong relationship with Oklahoma-94-2009 strain. Phylogenetic analysis indicated four different clusters of HPIV-2 strains (G1-4). Twenty-three amino acid substitutions were recorded for Riyadh 105/2009, from which four are unique. The majority of substitutions (n=18) had changed their amino acids characteristics. By analyzing the effect of the recorded substitutions on the protein function using SIFT program, only two located at positions 360 and 571 were predicted to be deleterious.

**Conclusions:**

The presented changes of Riyadh 105/2009 strain may possess potential effect on the protein structure and/or function level. This is the first report that describes partial characterization of Saudi HPIV-2 strain.

## Findings

Human parainfluenza type 2 virus (HPIV-2) is one of the important viruses that infect the human respiratory tract causing croup and pneumonia [1]. It is an enveloped non-segmented negative single-stranded RNA virus that belongs to genus *Rubulavirus* of the family *Paramyxoviridae*. The virus genome is approximately 15 kb long and encodes seven structural proteins. The nucleocapsid (N), phosphoprotein (P), and large polymerase (L) encapsidate the genomic RNA, while hemagglutinin-neuraminidase (HN), fusion (F), and matrix (M) are envelope-associated proteins [2].

HN protein constitutes the prominent virus fringes responsible for binding to sialoconjugate receptors (hemagglutinating activity), and for enzymatic destruction of the receptors (neuramindase activity). Moreover, the stalk region of HN was proposed to contain the site which promotes cell fusion by F protein [3,4]. Extensive genetic and antigenic variations among HPIV-2 strains were attributed to HN gene diversity [5,6]. Two distinct clusters were suggested by phylogenetic analysis of HPIV-2 strains on the basis of complete HN gene sequence. However, this phylogenetic tree was inadequately informative due to the low number of analyzed sequences [5]. In this report, the complete HN sequence of first Saudi HPIV-2 strain was analyzed and compared to the corresponding sequences available in GenBank. A phylogenetic tree was constructed to determine the predictable origin of Saudi strain and the relationship between different strains circulating globally.

Nasopharyngeal aspirates of 180 children, hospitalized with acute respiratory disease in King Khalid University Hospital, Riyadh, were obtained as described before [7]. Sample collection was approved by the Ethical Committee of King Saud University. The aspirates were taken from patients, after getting an informed consent from their parents. Samples were chilled transported in Minimal Essential Medium supplemented with 500 U penicillin and 500 μg streptomycin per ml (Sigma, St. Louis, MO) to Virology Research Laboratory, King Saud University. After pulse-vortexing and centrifugation at 3000 rpm for 10 min, 140 μl aliquots of the clarified supernatants were utilized for RNA extraction using QIAamp viral RNA extraction kit (Qiagen, Hilden, Germany) according to the manufacturer`s guidelines. Detection of HPIV-2 RNA was accomplished by amplification of 508 bp fragment of HN gene using One-step RT-PCR kit (Qiagen) and the primer set HPIV2-cDNA/HPIV2-rev (Table 1). Positive reactions were confirmed by the amplification of an internal 204 bp fragment of the PCR amplicon in a nested PCR using TopTaq Master Mix Kit (Qiagen), and the primer set HPIV2-nF/HPIV2-nR (Table 1). Products of both reactions were analyzed, along with 100 bp DNA ladder (Qiagen), in 1.5% agarose gel stained with ethidium bromide.

**Table 1:** Oligonucleotide primers used in the study

**PCR reaction / Primer (Polarity)**	**Sequence (5′to 3′)**	**Position**^**a**^	**PCR product**
**Primary**			508 bp
HPIV2-cDNA (+)	AACAATCTGCTGCAGCATTT	7437-7456	
HPIV2-rev (−)	ATGTCAGACAATGGGCAAAT	7925-7944	
**Nested**			204 bp
HPIV2-nF(+)	CCATTTACCTAAGTGATGGAAT	7479-7500	
HPIV2-nR(−)	GCCCTGTTGTATTTGGAAGAGA	7661-7682	
**Sequencing**			
HPIV2-HN-F1(+)	CGAACCCTTAAGGTGTCGTAACGTC	6648-6672	886 bp
HPIV2-HN-R1 (−)	GGTATAGCAGTGACTGAACAGC	7512-7533	
HPIV2-HN-F2(+)	GACCCATTGGTGTTACACTCACAATG	7348-7373	840 bp
HPIV2-HN-R2(−)	GTTGCATTGCATGGCATGACTC	8166-8187	
HPIV2-HN-F3(+)	GGTACCGTCCTATCAAGTTCC	8137-8157	426 bp
HPIV2-HN-R3 (−)	GAATCTGGAGTCTTCATTCAATAG	8539-8562	

^a^ Nucleotide positions based on the complete genome sequence of HPIV-2 strain Greer; GenBank accession number AF533012.

Only one sample (0.56%), collected from two-year old female child, was confirmed positive for HPIV-2. The sample, designated Riyadh 105/2009, was used for sequencing and phylogenetic analysis of the complete HN gene. Sequencing strategy involved the amplification of three overlapping fragments, that flank the entire HN gene sequence, using FideliTaq™ RT-PCR Master Mix (GE Healthcare, Buckinghamshire, UK) and sequencing primer sets (Table 1). Specific RT-PCR products were purified from agarose gel using Illustra GFX PCR DNA Kit (GE Healthcare), and were sequenced on both strands (GenArt, Rosensburg, Germany). Nucleotide sequences were edited and assembled using Bioedit Biological Sequence Alignment Editor (Ibis Biosciences, Carlsbad, CA). Complete nucleotide and deduced amino acid sequences of HN gene of Riyadh 105/2009 strain were deposited in GenBank under the accession number HM460888. Multiple alignments with 17 HPIV-2 complete HN sequences available in GenBank (Table 2) were performed using the Clustal W algorithm of Megalign program, Lasergene software. Divergence analysis and tree construction were conducted using the same software, with 1,000 bootstrap replicates. The effect of non-synonymous mutations on the protein function was predicted using SIFT program ( http://sift.bii.a-star.edu.sg).

**Table 2:** Amino acid differences among the selected HPIV-2 strains as compared to the Saudi strain Riyadh 105/2009

**Strain**^**a**^	**Country/Year**	**18**	**48**	**50**	**54**	**57**	**67**	**114**	**139**	**164**	**175**	**195**	**201**	**319**	**332**	**344**	**345**	**351**	**360**	**367**	**376**	**443**	**482**	**571**
Riyadh 105/2009^b^	Saudi Arabia/2009	K	A	P	N	D	V	T	K	H	S	T	A	S	T	E	R	G	Y	I	Q	H	Q	P
86-391	Japan/2004	R	V	S	D	E	I	A	E	N	I	A	S	T	K	K	Q	S	N	V	H	Y	R	L
76-86	Japan/2004	R	V	S	D	E	I	A	E	N	I	A	S	T	K	K	Q	S	N	V	H	Y	R	L
4-80	Japan/2004	R	V	S	D	E	I	A	E	N	I	A	S	T	K	K	Q	S	N	V	H	Y	R	L
TC-6482	Japan/2004	R	V	S	D	E	I	A	E	N	I	A	S	T	K	K	Q	S	N	V	H	Y	R	L
TC-6558	Japan/2004	R	V	S	D	E	I	A	E	N	I	A	S	T	K	K	Q	S	N	V	H	Y	R	L
62-M786	Japan/2004	R	V	S	D		I			N	I						Q	N	N	V	H	Y		
Greer-J1	Japan/2007	R	V	S	D		I			N	I				K		Q	S	N	V	H	Y		
Toshiba	Japan/1990	R	V	S	D		I			N	I				K		Q	S	N	V	H	Y		
London	UK/1990	R	V	S	D		I			N	I				K		Q	S	N	V	H	Y		
Lyon-26056-1997	France/1997	R	V	S	D	E	I	A	E	N	I	A	S	T	K	K	Q	N	N	V	H	Y	R	L
Lyon-18620-2001	France/2001	R	V	S		E	I	A	E		I	A	S	T	K	K	Q	S	N	V	H	Y	R	L
Lyon-20283-2001	France/2001	R	V	S		E	I	A	E		I	A	S	T	K	K	Q	S	N	V	H	Y	R	L
Lyon-20435-2001	France/2001	R	V	S		E	I	A	E		I	A	S	T	K	K	Q	S	N	V	H	Y	R	L
V9412-6	USA/1999	R	V	S		E	I	A	E		I	A	S	T	K	K	Q	S	N	V	H	Y	R	L
Oklahoma-94-2009	USA/2009	R	V	S																		Y		
Oklahoma-283-2009	USA/2005	R	V	S		E	I	A	E		I	A	S	T	K	K	Q	S	N	V	H	Y	R	L
Oklahoma-3955-2005	USA/2005	R	V	S	D	E	I	A	E	N	I	A	S	T	K	K	Q	S	N	V	H	Y	R	L

^a^ Accession numbers of the strains used in analysis –in order– are: Genbank HM460888, AB189949, AB189950, AB189951, AB189952, AB189953, AB189948, AB367954, X57559, D00865, DQ072589, DQ072586, DQ072587, DQ072588, AF213352, JF912194, JF912195, and JF912196.

^b^ Blank cell indicates an amino acid identical to that of strain Riyadh 105/2009.

As expected, the results of sequence analysis revealed a single ORF of 1716 nucleotides that encodes a polypeptide of 571 amino acids [8]. Multiple sequence comparisons between Riyadh 105/2009 strain and the different global strains showed that the homology ranges from 91.8% to 99.1% and from 93.9% to 99% on the levels of nucleotide and deduced amino acids, respectively. A close relationship between the Saudi strain and Oklahoma-94-2009 strain (GenBank Accession number JF912194) was observed with only 16 nucleotide (0.9%) and 4 amino acid (0.7%) variations. This relationship may propose a common ancestral origin of both viruses. Seven unique nucleotide substitutions (0.41%) were found in Riyadh 105/2009 strain (G53A, A54G, T143C, T148C, T1327C, T1383, and G1401). As a result, four amino acids (0.53%) at positions 18, 48, 50, and 443 were changed.

**Figure 1:**
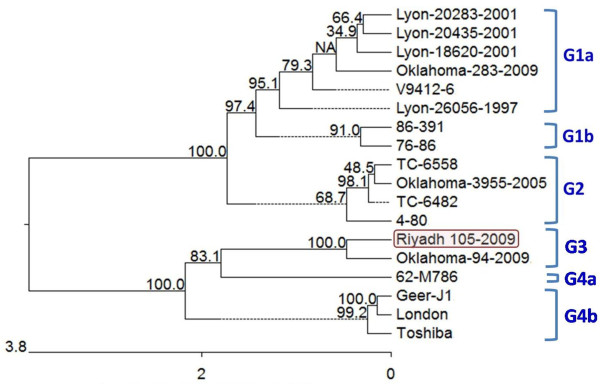
**Phylogenetic analysis based on the complete HN gene nucleotide sequence of the different HPIV-2 strains circulating globally.** Tree was constructed using Megalign program of Lasergene software (DNASTAR) with 1000 bootstraps. HPIV-2 strains included in the analysis were grouped in four clusters (G1-4). Each of clusters G1 and G4 were split into two subclusters (a and b). Riyadh 105/2009 strain was indicated by a red frame, and was grouped with Oklahoma-94-2009 strain in cluster G3. Scale bar indicates number of nucleotide substitution per site.

Phylogenetic analysis of the different worldwide strains of HPIV-2 indicated four different clusters (G1-4). Clusters G1 and G4 were further subdivided into subclusters a and b (Figure 1). Both Riyadh 105/2009 and Oklahoma-94-2009 strains were grouped together in cluster G3. The nucleotide and deduced amino acid sequence comparisons, between Riyadh 105/2009 strain and the different strains of each group, further established this classification. For instance, the homology between Riyadh 105/2009 and G1 viruses ranged from 91.8-92.7% (nucleotides), and 93.9-94.8% (amino acids), while for G2 and G4 viruses, the homology was 93–93.2%, and 96.2-96.4% on the nucleotide level, and 95.1-95.3%, and 96–96.2% on the amino acid level, respectively. This Phylogenetic differentiation correlates well with the tree constructed by Terrier et al. [5], in which two clusters were defined. The first cluster included G1 and G2 strains of the conducted phylogram, while the second contained strains of G4 cluster. G3 cluster strains were not included in that study due to their recent identification. It may be concluded that new variant strains are continuing to emerge, and therefore, further updating of this phylogram may be required upon identification of more strains on spatial and temporal basis.

Analysis of the deduced amino acid sequence alignment identified 23 positions of potential variation between Riyadh 105/2009 and other international strains (Table 2). Nineteen of these positions were shared with the other G3 virus (Oklahoma-94-2009), while 9–10, and 0–2 positions were only common with G4, and G1-2 viruses, respectively. The majority of the recorded substitutions (n=18) changed the amino acid characteristics on the level of charge, polarity, and/or hydropathy. However, the effect of these substitutions on the antigenic properties of the mature HN protein could not be concluded, since positions of the antigenic sites are not yet determined for HPIV-2. Aligning the positions of amino acid substitution of Riyadh 105/2009 strain with their counterparts in HPIV-3 HN protein identified a single residue (Q345R) that may be located in a distinct antigenic site [9].

Studying the effect of amino acid change on the protein function using SIFT program [10], outlined that all of the substitutions are well tolerated; except the change of asparagine (N) to tyrosine (Y) at position 360, and leucine (L) to proline (P) at position 571 that were predicted to affect the protein function with a score of 0.02 and 0.00, respectively. These two residues locate in the globular head region that carries both haemagglutinating and neutamindase activities [11]. However, the extent of their effect on the protein function would be correctly concluded when the three-dimensional structure of HN protein becomes available, and the amino acids crucial for both activities are determined.

In conclusion, the complete HN gene sequence of the first HPIV-2 Saudi strain (Riyadh 105/2009) was analyzed and compared to the corresponding sequences/strains at GenBank. Phylogenetic analysis of these strains allowed allocation of four separate clusters G1-G4. Saudi strain was more relevant to Oklahoma-94-2009 strain, which may propose a common ancestral origin. The majority of the amino acid substitutions observed in Riyadh 105/2009 strain changed the properties of their respective amino acids. However, the effect of these substitutions on the protein structure and function requires more detailed studies.

## Competing interest

The authors declare that they have no conflicts of interest.

## Authors’ contributions

FNA and HMA developed the concept, designed experiments and interpreted the results. MSS and HMA carried most of the experiments. FNA supervised the process of sample collection by trained nurses and helped with the experiments. All of the authors read and approved the manuscript.
